# Hepatocyte KCTD17-mediated SERPINA3 inhibition determines liver fibrosis in metabolic dysfunction-associated steatohepatitis

**DOI:** 10.1038/s12276-025-01499-w

**Published:** 2025-08-01

**Authors:** Yelin Jeong, Ah-Reum Oh, Young Hoon Jung, Kyung Hee Jung, Seongju Lee, Michele Carrer, Sang Bae Lee, Luca Valenti, Utpal B. Pajvani, KyeongJin Kim

**Affiliations:** 1 Department of Biomedical Sciences, College of Medicine, Incheon, Republic of Korea; 2 Program in Biomedical Science and Engineering, Incheon, Republic of Korea; 3 Research Center for Controlling Intercellular Communication, Incheon, Republic of Korea; 4 Department of Anatomy, College of Medicine, Inha University, Incheon, Republic of Korea; 5 Ionis Pharmaceuticals Inc., Carlsbad, CA USA; 6 Division of Life Sciences, Jeonbuk National University, Jeonju, Republic of Korea; 7 Department of Pathophysiology and Transplantation, Università degli Studi di Milano, Milan, Italy; 8 Precision Medicine, Biological Resource Center Unit, Fondazione IRCCS Ca’ Granda Ospedale Maggiore Policlinico, Milan, Italy; 9 Department of Medicine, Columbia University, New York, NY USA

**Keywords:** Metabolic disorders, Mechanisms of disease

## Abstract

Metabolic dysfunction-associated steatohepatitis (MASH) is a leading cause of chronic liver disease. Available therapies show inconsistent results on fibrosis, probably due to heterogeneity in disease trajectory or incomplete understanding of molecular determinants. Here we identified increased *KCTD17* levels in patients with MASH, and in dietary rodent models of MASH—such as those fed a diet high in palmitate, sucrose and cholesterol coupled with fructose-containing drinking water or a choline-deficient, l-amino acid-defined, high-fat diet—which showed an inverse correlation with the expression of serine protease inhibitor a3k (*SERPINA3* in humans, *Serpina3k* in mice). KCTD17 depletion increased *SERPINA3* levels and reduced liver fibrosis in mice fed a MASH-inducing diet by inhibiting Par2/TGFβ-mediated activation of hepatic stellate cells. Mechanistically, Kctd17 regulates *Serpina3k* expression by facilitating the ubiquitin-mediated degradation of Zbtb7b, which in turn diminishes Serpina3k secretion. Consequently, pharmacological inhibition of Kctd17 effectively reverses MASH-induced liver fibrosis. In summary, these findings underscore the therapeutic potential of targeting KCTD17 for the treatment of MASH-induced liver fibrosis.

## Targeting KCTD17 offers new hope for liver fibrosis

Metabolic dysfunction-associated steatotic liver disease (MASLD) affects about 25% of people worldwide. Around 20% of these cases progress to a more severe form, leading to liver damage and potentially requiring a liver transplant. Researchers studied a protein called KCTD17, which is involved in liver fibrosis. They found that reducing KCTD17 in mice with MASLD decreased liver damage. The study used mice fed a special diet to mimic human liver disease. The researchers used genetic techniques to lower KCTD17 levels and observed less liver scarring. They also discovered that KCTD17 affects another protein, Serpina3k, which helps protect the liver. Increasing Serpina3k levels reduced liver damage in mice. These findings suggest that targeting KCTD17 could be a new way to treat liver fibrosis. In the future, it could lead to new treatments for people with severe liver disease.

This summary was initially drafted using artificial intelligence, then revised and fact-checked by the author.

## Introduction

Metabolic dysfunction-associated steatotic liver disease (MASLD), previously known as nonalcoholic fatty liver disease, is estimated to affect approximately 25% of the global population^1–5^. Approximately 20% of MASLD cases progress to metabolic dysfunction-associated steatohepatitis (MASH), which is characterized by inflammation, fibrosis and liver cell injury^6,7^. This progression increases the risk of further development to liver cirrhosis and hepatocellular carcinoma, eventually requiring liver transplantation to prevent liver-related death^8,9^. While a therapeutic was recently approved for treatment of patients with moderate-to-advanced liver fibrosis, only a minority of treated individuals showed fibrosis regression^10–12^. As such, novel therapeutic approaches are still needed for patients with advanced MASH.

Liver fibrosis in MASH develops through cellular networks involving crosstalk with various liver cells, including hepatocytes, Kupffer cells and hepatic stellate cells (HSCs). Injured hepatocytes release profibrogenic mediators, such as secreted phosphoprotein 1 (SPP1), monocyte chemoattractant protein 1 (MCP-1) and Indian hedgehog (IHH), which recruit liver macrophages and trigger HSC activation^13–15^. Activated HSCs produce collagen and other extracellular matrix components, contributing to liver fibrosis^16^. KCTD17 is a member of the potassium channel tetramerization domain-containing protein family, characterized by a BTB/POZ domain involved in cell cycle, proliferation, gene regulation, intracellular signaling and cytoskeleton organization^17–19^. We previously observed that KCTD17 serves as an adaptor for substrate recruitment to Cul3-RING E3 ubiquitin ligases to regulate hepatic steatosis^20,21^ by regulating sterol regulatory element-binding protein 1c (Srebp1c) and carbohydrate-responsive element-binding protein (Chrebp) activity in obese mice and patients with MASLD^22^. However, whether KCTD17 may influence hepatic fibrosis was unknown.

In this study, we found that hepatocyte-specific Kctd17 loss-of-function or treatment with *Kctd17*-directed antisense oligonucleotide (ASO) reduced liver fibrosis in MASH mice fed a MASH-provoking diet. Hepatocyte Kctd17 induced ubiquitin-mediated protein degradation of zinc finger and BTB domain-containing protein 7b (Zbtb7b), which led to diminished expression and secretion of serine protease inhibitor a3k (Serpina3k), a member of the extracellular serpin family A member 3 and homologous to human SERPINA3^23,24^. Conversely, forced expression of hepatocyte Serpina3k or SERPINA3 alleviated liver fibrosis by reducing HSC activity through protease-activated receptor 2 (Par2)-mediated TGFβ1 signaling. These findings suggest that KCTD17 is a therapeutic target for liver fibrosis, and Serpina3k/SERPINA3 could serve as a potential biomarker of MASH.

## Materials and methods

### Animals

Eight-week-old Cre^−^ control or *L-Kctd17* mice were created by transducing male Rosa26-LSL-Cas9 knockin mice (#026175, Jackson Laboratory)^25^ with either AAV8-TBG-Cre or AAV8-U6-Kctd17-sgRNA-TBG-Cre at a dose of 1.5 × 10^11^ genome copies per mouse. Mice were fed either a high-fat and high-sucrose diet with 1.25% added cholesterol (FPC, TD.160785) for 16 weeks or an l-amino acid rodent diet with 60 kcal% fat with 0.1% methionine and no added choline (CDAHFD, A06071302) for 12 weeks. C57BL/6N were purchased from Orient Bio Inc., and 8-week-old AAV8-TBG, AAV8-TBG-mSerpina3k, AAV8-TBG-hSERPINA3 mice were produced by transduction with either AAV8-TBG, AAV8-TBG-mSerpina3k-GFPSpark or AAV8-TBG-hSERPINA3-GFPSpark with a dose of 3 × 10^11^ genome copies per mouse and then fed CDAHFD for 12 weeks. Mice were fasted for 16 h and then refed either FPC or CDAHFD for 4 h before euthanasia. Mice were housed four to five animals per cage, with a 12-h light/dark cycle in a temperature-controlled environment. All animal experiments were approved by Inha University and the Inha University Institutional Animal Care and Utilization Committee.

### Bioinformatics

Human and mouse transcriptomic data are available via Gene Expression Omnibus under the accession numbers GSE167523, GSE126848, GSE130970, and GSE137449. Heat maps were built using GENE-E (Broad Institute); the depth of shading at the correlation matrices (correlogram) indicates the magnitude of the correlation (Spearman’s rho). Correlograms and interaction networks were generated using RStudio (R Consortium), with positive and negative correlations represented in blue and red, respectively. Only correlations with Spearman’s rho >0.5 or <−0.5 are displayed in the interaction network.

### Constructs

Twenty-nucleotide single-guide RNA (sgRNA) sequences for Kctd17 were designed using the CRISPR design tool, cloned into the LentiCRISPRv2 or pAAV-sgRNA-TBG-Cre^22^. AAV-TBG-mSerpina3k-GFP or AAV-TBG-hSERPINA3-GFP was first cloned into the pENN-AAV-TBG vector from pCMV3-Serpina3kGFPSpark or pCMV3-SERPINA3GFPSpark (SinoBiological) and then was co-transfected with pAAV-RC2/8 and pAAV-Helper vector into AAVpro 293T cells (Takara Bio) to generate recombinant AAV and concentrated by Amicon Ultra 0.5-ml centrifugal filters (Merck).

### Cell culture studies

AML12, Hepa1c1c7, HSC-T6 and HEK293T cells were cultured in Dulbecco’s modified Eagle medium (Welgene) supplemented with 10% fetal bovine serum (Gibco) and 1% antibiotic antimycotic (Welgene). Lentivirus encoding Tet-on *Kctd17* short hairpin RNAs was produced by co-transfection of LentiX-293T cells (Takara) with the lentiviral vectors psPAX2 and pMD2.G. Viral supernatants were then transferred into Hepa1c1c7 cells with 8 μl/ml polybrene (SantaCruz Biotechnology), which were selected with puromycin (Thermo Fisher Scientific) and knocked down by doxycycline (Dox) treatment (Sigma-Aldrich). LX2 cells were cultured in Dulbecco’s modified Eagle medium supplemented with 2% fetal bovine serum and 1% antibiotic antimycotic. HSC-T6 or LX2 cells were stimulated with recombinant human TGFβ1 (Peprotech) and human plasma-derived SERPINA3 (α-ACT) (Sigma-Aldrich). All cells were incubated in 5% CO_2_ at 37 °C.

### Transfection and conditioned media

AML12, Hepa1c1c7 and HEK293T cells were transfected with plasmid expressing Kctd17, Serpina3k, SERPINA3 and Zbtb7b using Lipofectamine 3000 (Life Technologies) according to the manufacturer’s instructions. For conditioned media (CM), after 48 h transfection, serum-free media were changed, and the supernatant was transferred with or without TGFβ1 to the HSC-T6 or LX2 cell line. Ad-shControl and Ad-shKctd17 adenoviruses have been previously described^26^. In brief, Hepa1c1c7 cells were treated with Ad-shControl or Ad-shKctd17 adenovirus at a multiplicity of infection of 20 and treated with 2.5–20 μM MG-132 (Merck) for 6–16 h for inhibition of proteasomal degradation.

### RNA interference

LX2 cells were transfected with scramble small interfering RNA (siRNA, control) or siPar2 (Bioneer) using Lipofectamine 3000 according to the manufacturer’s instructions. The antisense sequences of siRNAs were as follows: siPar2 #1: 5′- GAC UUG UGU GUA AGA CUC A -3′ and siPar2 #2: 5′- GAG UUG GGA UUG GAC AGU A -3′. After 48 h of transfection, cells were stimulated as described above.

### RNA extraction and quantitative PCR

Total RNA was extracted from the liver tissue or cells by TRIzol (Invitrogen), and the quality and concentration of the RNA was assessed by measuring the absorbance at 260 and 280 nm using a NanoDrop spectrophotometer (Thermo Fisher Scientific). The cDNA was synthesized using a High-Capacity cDNA Reverse Transcription kit (Applied Biosystems), followed by quantitative RT-PCR with Power SYBR Green PCR master mix (Takara Bio) in a CFX Opus 96 Real-Time PCR detection system (Bio-Rad). The primer sequences are listed in Supplementary Table 1.

### Western blots and immunoprecipitation

Liver protein lysates were extracted using a modified radio immunoprecipitation assay (RIPA) buffer, and the protein concentration was measured using the bicinchoninic acid assay (Thermo Fisher Scientific). Proteins were separated by electrophoresis on 8–12% Tris gels and transferred to 0.2-μm polyvinylidene fluoride blotting membranes (GE Healthcare Life Science). The membranes were incubated with the 1:2,000 primary antibody in TBST (Tris-buffered saline (TBS) with 0.1% Tween 20) containing 3% bovine serum albumin (BSA) at 4 °C overnight, and then incubated with the 1:2,000 secondary antibody coupled to horseradish peroxidase, and proteins were detected using the Pierce ECL Western Blotting Substrate (Thermo Fisher Scientific) or ECL Prime Western Blotting Detection Reagent (Cytiva). Immunoblots were conducted on three to seven samples randomly chosen within each experimental cohort and antibodies against HA-tag (#3724) Myc-tag (#2276), GFP (#2956), β-actin (#4970), Smad2/3 (#8685), p-Smad2/3 (#8828), ERK (#4695S), p-ERK (#4370S) and α-SMA (#19245) from Cell Signaling; Flag-tag (#F1804) from Sigma; Serpina3k (#55480-1-AP) from Proteintech; SERPINA3 (#A1021) from Abclonal; Zbtb7b (#NBP1-76516) from NOVUS; and Kctd17 (RB8456) from own manufacturing. For immunoprecipitation, Hepa1c1c7 or HEK293T cells were co-transfected with the indicated plasmids for 48 h and then lysed. Lysates (500–800 μg) were then incubated overnight at 4 °C with 10 μl of EZview Red ANTI-FLAG M2 Affinity Gel (F2426, Sigma) and EZview Red Anti-HA Affinity Gel (E6779, Sigma). The beads were washed seven to eight times with cold RIPA buffer, and then 2× sample buffer was added for immunoblotting.

### Ubiquitination assays

Hepa1c1c7 cells were co-transfected with the indicated plasmids for 48 h and then denatured in TBS (Biosesang) and mixed with 20% sodium dodecyl sulfate (Biosolution). After heating for 15 min at 100 °C, the lysate was added to TBS containing 1% NP40 (Sigma-Aldrich) and centrifuged at 15,000 rpm for 10 min at 4 °C. Protein concentration was measured, and 500–800 μg of the lysate was used for immunoprecipitation and immunoblotting.

### Hematoxylin–eosin (H&E) staining and immunohistochemistry

Human tissue array was purchased from XenoTech. The tissues were immediately fixed with 3.7% paraformaldehyde at 4 °C overnight, embedded in paraffin and sectioned. The sections were then stained with H&E or incubated with KCTD17 primary antibody (1:100, Thermo Fisher Scientific) or Serpina3k (1:100, Proteintech) at 4 °C overnight, followed by three washes with phosphate-buffered saline (PBS) buffer (3 min per wash). The sections were incubated with enhanced enzyme-labeled goat anti-rabbit IgG polymer (reagent 3, PV-9001, ZSGB-Bio) for 20 min at 37 °C and rinsed with PBS buffer three times (3 min per wash). The sections were visualized using 3, 3’-diaminobenzidine (DAB) (ZLI-9018; ZSGB-Bio) and were counterstained with hematoxylin. Images were captured using a microscope (Olympus DP80) and quantitated using ImageJ software from the National Institutes of Health (NIH).

### Alanine aminotransferase and aspartate aminotransferase

Alanine aminotransferase (ALT) and aspartate aminotransferase (AST) levels were measured in mouse serum to evaluate liver injury using the TECO Diagnostics ALT/SGPT colorimetric reagent or TECO Diagnostics AST/GOT LIQUID (KINETIC; Thermo Fisher Scientific, TMO). Human ALT levels were measured by DRI-CHEM MX500i (Fujifilm) with an automatic dry-chemistry analyzer, according to the manufacturer’s instructions.

### Enzyme-linked immunosorbent assay (ELISA)

Human serum was purchased from bioIVT, and SERPINA3/alpha-1-antichymotrypsin concentration in serum was analyzed using an ELISA kit (EH411RB, Invitrogen) following the manufacturer’s instructions.

### Immunofluorescence

Cells were fixed with 4% paraformaldehyde and permeabilized with 0.1% Triton X-100 in PBS. They were then blocked with 1% BSA in PBST (PBS with 0.1% Tween 20) and incubated overnight at 4 °C with primary antibodies against Zbtb7b and HA (1:500, Cell Signaling Technology) in 1% BSA in PBST. They were then incubated with secondary antibody (1:1,000, anti-rabbit Alexa Fluor 488 (#A-21206, Invitrogen) and anti-mouse Alexa Fluor 594 (#A-21203, Invitrogen)) in 1% BSA for 1 h at room temperature in the dark. The cells were further incubated with 0.1–1 μg/ml 4′,6 diamidino-2-phenylindole (MBD0015, Sigma-Aldrich) for counterstaining and mounted in VECTASHIELD (H-1700, Vector Laboratories). Images were captured using fluorescence microscopy (U-HGLGPS light guide-coupled illumination system, Olympus DP80) and quantified using ImageJ software from the NIH.

### Sirius red staining

Liver paraffin sections were deparaffinized and rehydrated with distilled water. Collagen content was detected using a Picro-Sirius red staining solution (Polysciences) according to the manufacturer’s protocol, and mounted in VECTASHIELD (H-5000-60, Vector Laboratories). Images were captured using a microscope (Olympus) and quantified using ImageJ software.

### ASOs

Control ASO, *Kctd17* ASO-1 and *Kctd17* ASO-2 have been previously described^22,27,28^. In brief, nonspecific control ASO, *Kctd17* ASO-1 (5′-AGG TAA TGA TTG TAG C-3′) and *Kctd17* ASO-2 (5′-AAG GTA ATG ATT GTA G-3′) were synthetized by Ionis Pharmaceuticals, diluted in 0.9% NaCl before injection and administered by intraperitoneal injection to male C57BL/6N mice at a dosage of 20–25 mg/kg body weight once weekly for 6–7 weeks before euthanasia.

### Statistical analysis

All data are shown as the mean standard error of the mean (s.e.m.). Differences between the two groups were calculated using a two-tailed Student’s *t*-test or one-tailed Wilcoxon rank-sum test. Analyses involving multiple groups were performed using one-way analysis of variance or Kruskal–Wallis tests. *P* < 0.05 was considered statistically significant.

## Results

### KCTD17 levels are increased in patients with MASH

**Fig. 1:**
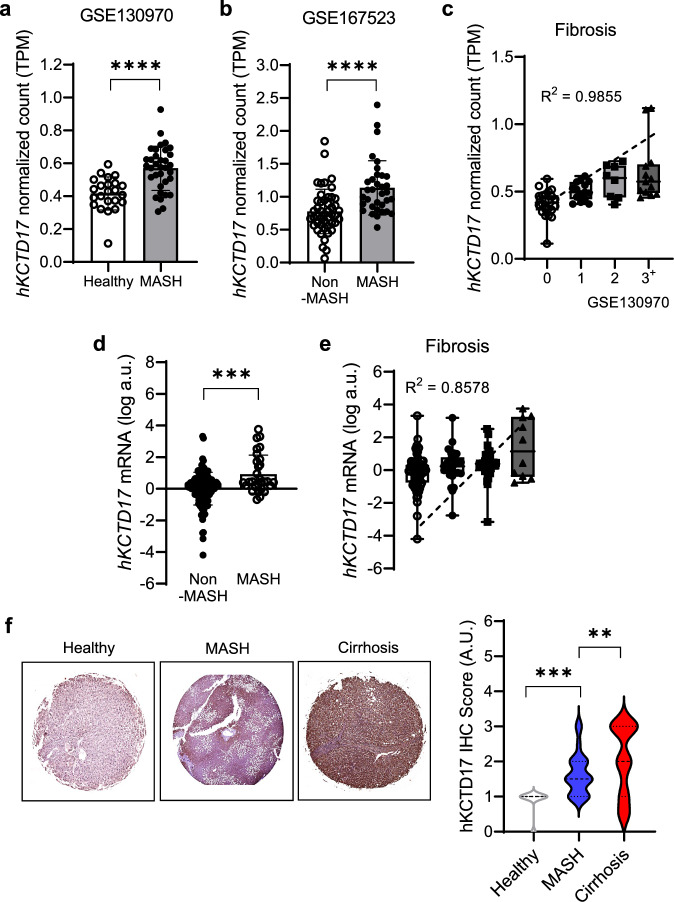
*KCTD17* levels are increased in patients with MASH. **a**, **b** *KCTD17* mRNA expression levels in the RNA-seq database from healthy or biopsy-proven non-MASH controls and MASH livers (**a**) GSE130970, healthy 23, MASH 36; **b** GSE167523, non-MASH 47, MASH 34). **c** Correlation between *KCTD17* and fibrosis score. TPM, transcripts per million (GSE130970). **d**, **e** *KCTD17* mRNA expression in liver biopsies (*n* = 158) (**d**) and correlation with the fibrosis score (**e**). **f** Representative immunohistochemical staining imaging of KCTD17 in human liver tissue samples (*n* = 25–73 per group). **P < 0.01, ***P < 0.001, ****P < 0.0001 compared with the healthy control according to a two-way analysis of variance. All data are shown as the means ± s.e.m.

To evaluate the clinical relevance of KCTD17, we compared RNA sequencing (RNA-seq) in healthy (GSE130970) or biopsy-proven non-MASH controls (GSE167523) with that from patients with MASH. We observed that *KCTD17* levels were elevated in the livers of patients with MASH and further correlated with fibrosis severity (Fig. 1a–c). Further analysis of liver biopsies from patients with MASH allowed us to assess liver *KCTD17* expression in relation to disease severity. Our results demonstrate a significant increase in *KCTD17* mRNA levels in patients with MASH compared with healthy individuals, with a strong positive correlation between *KCTD17* expression and liver fibrosis score across varying degree of MASH severity (Fig. 1d, e). In addition, the histopathological examination of liver biopsies revealed a progressive increase in KCTD17 protein levels in patients with MASH and cirrhosis (Fig. 1f). These findings indicate that KCTD17 expression escalates in the liver in correlation with the severity of MASH, suggesting its pivotal role in driving MASH pathogenesis.

#### Hepatocyte-specific Kctd17 depletion alleviates liver fibrosis in mouse models of MASH

**Fig. 2:**
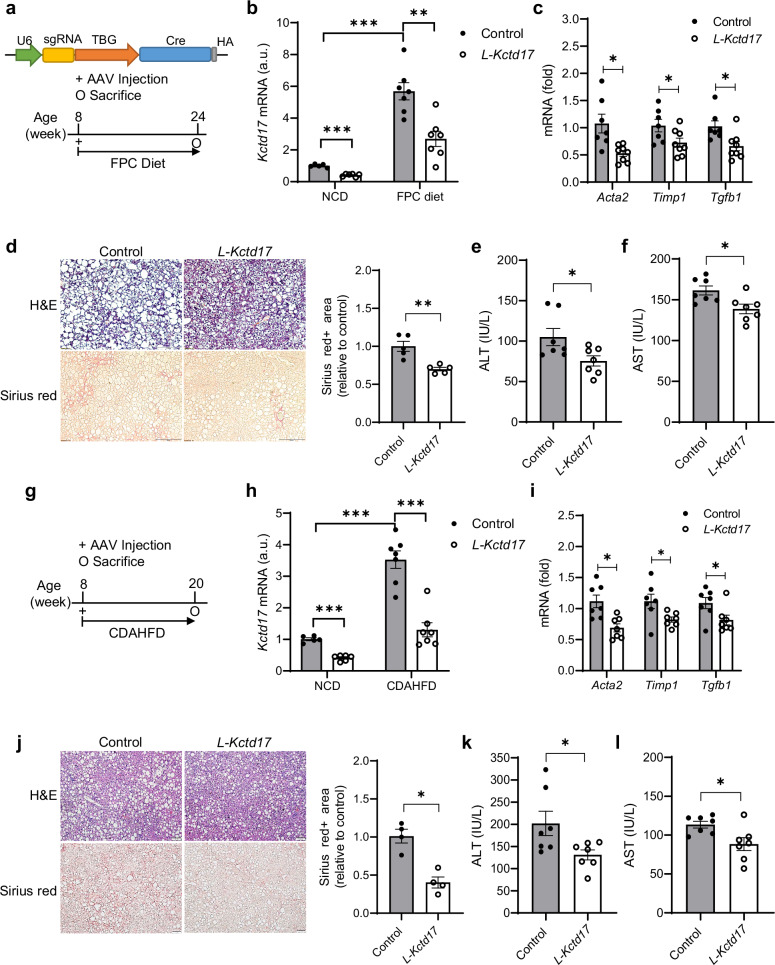
Hepatocyte-specific *Kctd17* depletion alleviates liver fibrosis in mouse models of MASH. **a** Cas9 knockin mice were transduced with AAV8-TBG-Cre (control) or AAV8-U6-sgKctd17-TBG-Cre (*L-Kctd17*) with an FPC diet and euthanized 16 weeks after transduction. **b** *Kctd17* mRNA expression in the liver from NCD or FPC diet-fed mice. **c** Liver gene expression (*n* = 7–8 per group). **d** H&E and Sirius red staining in histological sections of mouse liver, with respective quantification (*n* = 4 per group; scale bar, 200 μm; magnification, 20×). **e**, **f** ALT (**e**) and AST (**f**) levels in serum. **g** Control or *L-Kctd17* mice with CDAHFD were euthanized 12 weeks after transduction. **h** *Kctd17* mRNA expression in the liver from NCD or CDAHFD-fed mice (the NCD-fed mice data are identical to those shown in (**b**). **i** Liver gene expression (*n* = 7 and 8 per group). **j** H&E and Sirius red staining in histological sections of mouse liver, with respective quantification (*n* = 4 per group; scale bar, 100 μm; magnification, 20×). **k**, **l** Serum ALT (**k**) and AST (**l**) levels. *P < 0.05, **P < 0.01 compared with the control littermates according to a two-way analysis of variance. All data are shown as the means ± s.e.m.

These patient data indicate that KCTD17 may play a role in promoting liver fibrosis during MASH pathogenesis. To test this concept in mouse models, we first analyzed and observed elevated *Kctd17* expression in livers from fructose, palmitate, cholesterol and trans-fat (FPC) diet-fed mice^13,15,29^ (Fig. 2a, b). *Kctd17* was detected in both hepatocyte and nonparenchymal liver cells, but only hepatocyte *Kctd17* expression changed significantly in FPC diet-fed mice compared with livers from normal chow diet (NCD)-fed mice (Supplementary Fig. 1). To determine whether hepatocyte *Kctd17* is necessary for MASH-induced liver fibrosis, control or hepatocyte-specific *Kctd17*-knockout (*L-Kctd17*) mice^22^ were fed an FPC diet for 16 weeks (Fig. 2a). As anticipated, *L-Kctd17* mice showed lower liver *Kctd17* levels compared with control littermates (Fig. 2b) but also decreased expression of markers of HSC activity (Fig. 2c). These changes correlated with reduced hepatocellular injury and fibrosis, as indicated by lower liver Sirius red staining and decreased ALT and AST levels in serum (Fig. 2d–f). Notably, no obvious changes in body weight, liver, epididymal white adipose tissue (eWAT) weight or glucose levels were observed (Supplementary Fig. 2a-d).

We next tested another model of MASH-induced fibrosis in which mice were fed a choline-deficient, L-amino acid-defined, high-fat diet (CDAHFD)^30^ (Fig. 2g), which also showed high *Kctd17* mRNA and protein levels (Fig. 2h and Supplementary Fig. 2e). Similar to the results above, CDAHFD-fed *L-Kctd17* showed a reduction in fibrogenic markers, Sirius red staining in the liver, and serum ALT and AST levels (Fig. 2i–l), along with reduced liver staining of α-SMA and F4/80 (Supplementary Fig. 2f), supporting the involvement of Kctd17 in modulating HSC activity and inflammation in vivo. Again, there were no significant differences in body weight, liver, eWAT weight or glucose levels compared with control littermates (Supplementary Fig. 2g–j). These results suggest that increased hepatocyte Kctd17 in MASH is causal to liver injury and fibrosis.

#### Kctd17 reduces Serpina3k/SERPINA3 secretion in MASH

**Fig. 3:**
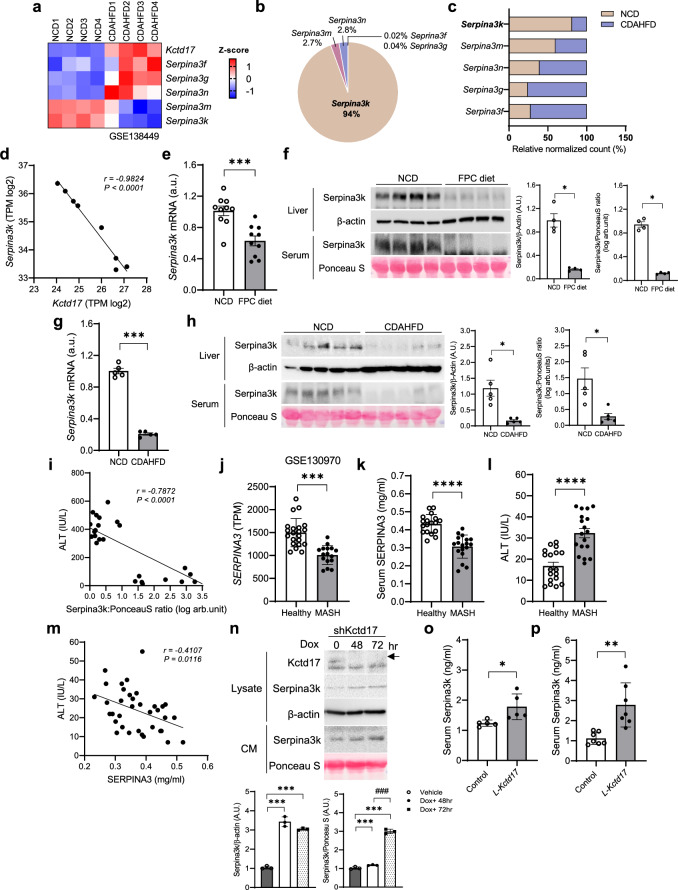
Kctd17 reduces Serpina3k/SERPINA3 secretion in MASH. **a** Heatmap from the RNA-seq database from NCD and CDAHFD-fed mice (GSE138449, *n* = 4 per group). **b**, **c** The relative abundance of Serpina3 subfamilies detected in the liver from NCD-fed C57BL/6 WT mice (**b**) and the relative normalized count of Serpina3 subfamilies in the RNA-seq database from NCD and CDAHFD-fed mice (**c**). **d** Correlation between Kctd17 and Serpina3k based on in RNA-seq from CDAHFD-fed C57BL/6 WT mice (GSE138449). **e**, **f** *Serpina3k* mRNA expression in the liver (**e**) and protein levels in the liver and serum (**f**) in FPC diet-fed WT mice. **g**–**i** *Serpina3k* mRNA expression in the liver (**g**) protein levels in the liver and serum (**h**) and correlation between Serpina3k and ALT (**i**). **j** *SERPINA3* expression levels in the RNA-seq database from healthy and MASH livers (GSE130970, healthy 21, MASH 16). **k**–**m** Serum SERPINA3 (**k**), ALT levels in healthy participants and patients with MASH (*n* = 18 per group) (**l**) and correlation between SERPINA3 and ALT (**m**). **n** Western blots from Hepa1c1c7 cells transduced with tet-on-shKctd17 and activated by 500 ng/ml Dox treatment for 48 h or 72 h and quantification. **o**, **p** Serpina3k level in serum by ELISA in FPC diet-fed (**o**) and CDAHFD-fed (**p**) *L-Kctd17* mice. *P < .05, **P < 0.01, ***P < 0.001, ****P < 0.0001 as compared with the indicated control; ^*###*^*P* < 0.001 as compared with that of Dox+ 48 h according to two-way analysis of variance. All data are shown as the means ± s.e.m.

To explore the mechanisms by which Kctd17 affects liver fibrosis, we analyzed liver transcriptomic profiles and screened for hepatocyte-secreted proteins in CDAHFD-fed wild-type (WT) mice^31^. This comprehensive analysis confirmed a significant increase in *Kctd17* expression, also revealed numerous differentially regulated genes and highlighted liver-specific secretory proteins that could potentially activate HSCs (Supplementary Fig. 3a), in particular a decrease in *Serpina3k* expression, the most abundant and altered Serpin3a-family gene^23^ (Fig. 3a–c). We confirmed the inverse correlation between *Kctd17* and *Serpina3k* expression in RNA-seq data from CDAHFD-fed mice (Fig. 3d). Thus, we observed lower liver and serum Serpina3k levels in FPC- or CDAHFD-fed mice (Fig. 3e–h), which were inversely correlated with serum ALT levels in CDAHFD-fed mice (Fig. 3i). Correspondingly, SERPINA3, the human homolog of Serpina3k, was found to be expressed at lower mRNA (Fig. 3j and Supplementary Fig. 3b) and serum levels (Fig. 3k) in patients with MASH (Supplementary Fig. 3c), which corresponded with ALT levels (Fig. 3l, m). These results suggest an inverse relationship between SERPINA3 levels and liver function in mice and humans that may be mediated by KCTD17. To test this, we used Dox-inducible *Kctd17*-knockdown Hepa1c1c7 cells and observed that suppression of Kctd17 enhanced Serpina3k levels in both lysates and CM in a time-dependent manner (Fig. 3n). Accordingly, we observed a recovery of Serpina3k levels in serum of FPC- or CDAHFD*-*fed *L-Kctd17* mice (Fig. 3o, p). Conversely, AAV8-TBG-Kctd17-transduced WT mice showed reduced liver *Serpina3k* (Supplementary Fig. 3d).

#### Restoration of hepatocyte Serpina3k/SERPINA3 improves liver fibrosis

**Fig. 4:**
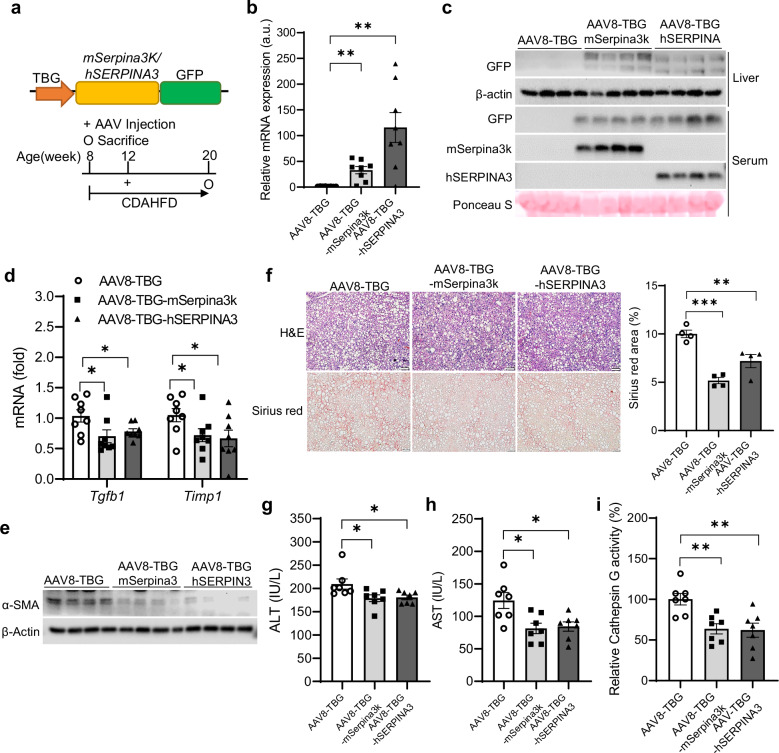
Restoration of hepatocyte Serpina3k/SERPINA3 improves liver fibrosis. **a** C57BL/6 WT mice were transduced with AAV8-TBG or AAV8-TBG-mSerpina3k-GFP (AAV8-TBG-mSerpina3k) or AAV8-TBG-hSERPINA3-GFP (AAV8-TBG-hSERPINA3) with CDAHFD and euthanized 12 weeks after transduction. **b**–**e** *Serpina3k* and *SERPINA3* mRNA levels (**b**) protein levels in the liver and serum (**c**), liver gene expression (*n* = 7–8 per group) (**d**) and α-SMA protein level in the liver (*n* = 4 per group) (**e**). **f** H&E and Sirius red staining in histological sections of mouse liver, with respective quantification (*n* = 4 per group; scale bar, 100 μm; magnification, 20×). **g**–**i** ALT (**g**) and AST (**h**) levels in serum and cathepsin G activity in the liver (*n* = 7 per group) (**i**). *P < 0.05, **P < 0.01, ***P < .001 compared with the AAV8-TBG control according to a two-way analysis of variance. All data are shown as the means ± s.e.m.

As endogenous levels of Serpina3k or SERPINA3 were pathologically reduced in the livers of FPC- or CDAHFD-fed WT mice and patients with MASH, we hypothesized that restoring Serpina3k/SERPINA3 could reverse hepatic steatohepatitis. In fact, we observed a significant reduction in *Serpina3k* expression and protein levels in the liver and serum as early as 4 weeks after starting CDAHFD feeding (Supplementary Fig. 4a–c). Thus, we introduced mouse or human SERPINA3 into WT mice at this time point (Fig. 4a–c) and found that both reduced HSC activity, associated with reduced liver Sirius red staining and plasma ALT and AST levels (Fig. 4d–h), without significant differences in body weight, liver, eWAT weight or glucose levels as compared with AAV8-TBG-transduced control littermates (Supplementary Fig. 4d–g). SERPINA3 inhibits the activity of serine protease, such as cathepsin G^32,33^, which is associated with diverse metabolic syndromes^34^. Therefore, we investigated whether cathepsin G is involved in hepatic fibrosis in MASH and is controlled by Serpina3k. Interestingly, cathepsin G activity was higher in both serum and livers from CDAHFD-fed mice compared with those given a NCD (Supplementary Fig. 5a), and treatments with cathepsin G promoted HSC activation (Supplementary Fig. 5b). Furthermore, cathepsin G activity was reduced in mSerpina3k and hSERPINA3-restored mice fed CDAHFD (Fig. 4i), suggesting a potential link between Serpina3k and cathepsin G activity. These findings demonstrate that forced hepatocyte expression of Serpina3k/SERPINA3 alleviates liver fibrosis and inhibits cathepsin G activity.

#### Kctd17 inhibits Zbtb7b-mediated Serpina3k expression

**Fig. 5:**
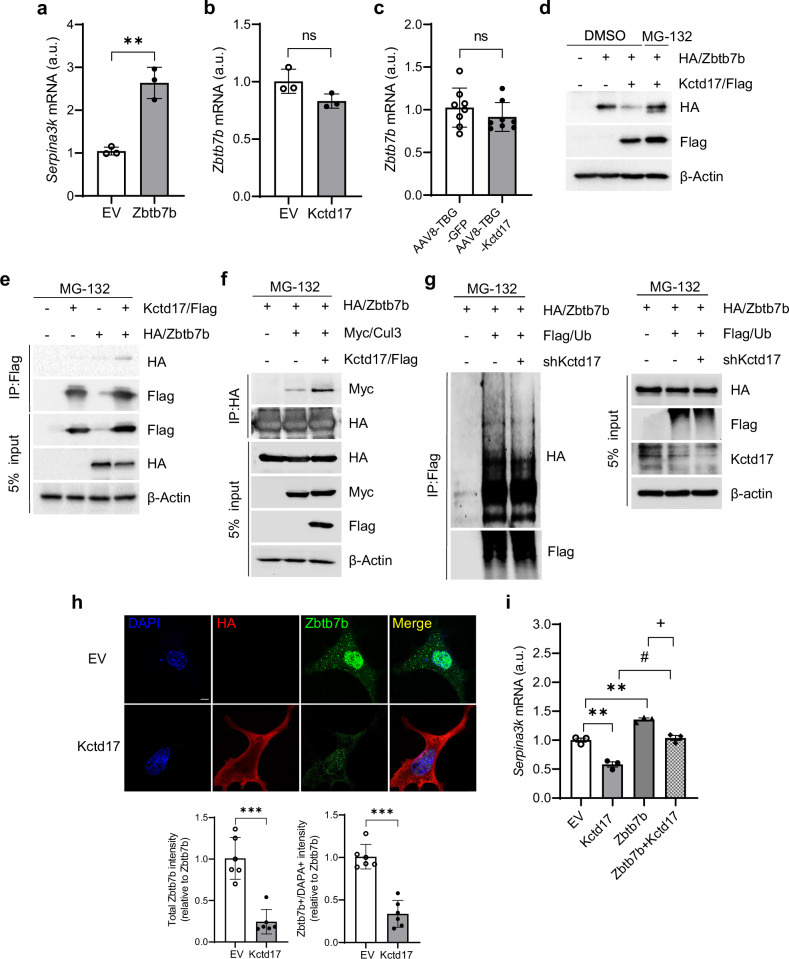
Kctd17 inhibits Zbtb7b-mediated Serpina3k expression. **a** *Serpina3k* mRNA expression from Hepa1c1c7 cells transfected with HA/Zbtb7b. **b** *Zbtb7b* expression from Hepa1c1c7 cells transfected with HA/Kctd17. **c** *Zbtb7b* expression in the liver from FPC diet-fed AAV8-TBG or AAV8-TBG-Kctd17 mice (*n* = 8 per group). **d** Western blots in Hepa1c1c7 cells transfected with HA/Zbtb7b and Kctd17/Flag with or without MG-132. **e**, **f** Co-immunoprecipitation of HA/Zbtb7b, Kctd17/Flag (**e**) and Myc/Cul3 (**f**) in Hepa1c1c7 cells with MG-132. **g** Zbtb7b ubiquitination in 3x Flag/Ub and Kctd17/Flag-transfected Hepa1c1c7 cells. **h** Immunofluorescent staining in EV or HA/Kctd17-transfected Hepa1c1c7 cells (*n* = 6 per group; scale bar, 10 μm; magnification, 60×). **i** *Serpina3k* mRNA expression from Hepa1c1c7 cells transfected with HA/Zbtb7b and/or Kctd17/Flag as indicated. **P < 0.01, ***P < 0.001 compared with the indicated EV control, ^*#*^*P* < 0.05 compared with Kctd17, ^*+*^*P* < 0.05 compared with Zbtb7b according to a two-way analysis of variance. All data are shown as the means ± s.e.m.

We next explored potential regulators of the proposed Kctd17–Serpina3k/SERPINA3 axis in MASH. We focused on Zbtb7b, which has been identified as a transcription activator of *SERPINA3* in humans^35^. Furthermore, we confirmed that Zbtb7b upregulated *Serpina3k* mRNA expression in murine hepatic cells (Fig. 5a). Intriguingly, although *Zbtb7b* mRNA was unchanged by Kctd17 manipulation both in vitro (hepatic cell lines transfected with Kctd17) and in vivo (livers from AAV8-TBG-Kctd17-transduced mice), we found consistent reductions in Zbtb7b protein, which was reversed by inhibition of proteasomal degradation (Fig. 5b–d). These data imply that Kctd17 regulates Zbtb7b protein stability and degradation. Indeed, we found that Kctd17 interacted with Zbtb7b and augmented the formation of the Zbtb7b–Cul3 ligase complex (Fig. 5e, f). As such, acute knockdown of Kctd17 led to decreased Zbtb7b ubiquitination, while Kctd17 overexpression lowered both cytosolic and nuclear levels of Zbtb7b (Fig. 5g, h) and, thus, *Serpina3k* transcription (Fig. 5i). Overall, these findings revealed that Kctd17 represses *Serpina3k* expression through proteasome-mediated degradation of Zbtb7b.

#### Serpina3k/SERPINA3 attenuates HSC activity through Par2-mediated TGFβ signaling pathways

**Fig. 6:**
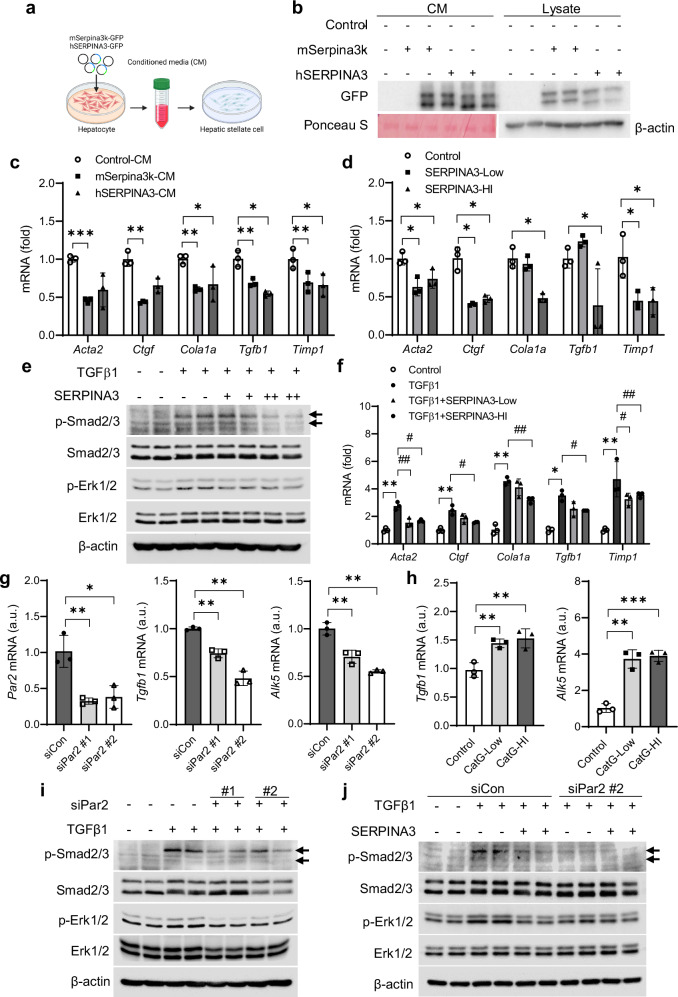
Serpina3k/SERPINA3 attenuates HSC activity through Par2-mediated TGFβ signaling pathways. **a** CM produced from AML12 cell transfected with plasmids expressing control, mSerpina3k-GFP or hSERPINA3-GFP after 48 h were applied to HSC-T6 cells. **b** Western blots of CM and cell lysates from transfected AML12 cells. **c** Fibrogenic gene expression by treatment of CM in HSC-T6 cells. **d** Fibrogenic gene expression by treatment with human plasma-derived SERPINA3 in LX2 cells. **e** Western blots of p-Smad2/3 and p-Erk1/2 after co-treatment with SERPINA3 (from human serum) with TGFβ1 (10 ng/ml) for 1 h. **f** Fibrogenic gene expression after co-treatment with SERPINA3 (from human plasma) with TGFβ1 (10 ng/ml) for 24 h in LX2 cells. **g** *Par2*, *Tgfb1* and *Alk5* mRNA levels in LX2 cells transfected with siPar2. **h** *Tgfb1* and *Alk5* mRNA levels after treatment with cathepsin G (50 or 100 ng/ml) for 24 h. **i** Western blots of p-Smad2/3 and p-Erk1/2 after treatment with TGFβ1 with or without silencing of Par2. **j** Western blots of p-Smad2/3 and p-Erk1/2 after co-treatment with TGFβ1 and SERPINA3 with or without silencing of Par2 in LX2 cells. *P < 0.05, **P < 0.01, ***P < 0.001 compared with the indicated control, ^*#*^*P* < 0.05, ^*##*^*P* < 0.01 compared with TGFβ1 according to a two-way analysis of variance. All data are shown as the means ± s.e.m.

To explore the molecular mechanisms by which hepatocyte-derived SERPINA3 or Serpina3k mitigates liver fibrosis, we produced CM from AML12 cells transfected with control, mSerpina3k-GFP or hSERPINA3-GFP, which were then applied to HSC-T6 rat HSCs (Fig. 6a, b). This CM-mediated treatment with mSerpina3k or hSERPINA3 reduced HSC activity, relative to control CM (Fig. 6c). Similar results were replicated with treatments with human plasma-derived SERPINA3, known as α-antichymotrypsin (α-ACT), in LX2 cells (Fig. 6d), suggesting that secreted Serpina3k/SERPINA3 defuses liver fibrosis by reducing HSC activation, perhaps by reducing TGFβ activity, as indicated by phosphorylation of Smad2/3 and Erk1/2 (Supplementary Fig. 6). These effects were more pronounced at high doses of human plasma-derived SERPINA3 (Fig. 6e, f).

We next considered whether these findings could be linked to our observation of reduced cathepsin G activity in hSERPINA3- or mSerpina3k-overexpressing mice (Fig. 4i), perhaps through Par2, a downstream substrate activated by cathepsin G^36,37^. Par2 is known to enhance HSC activation and liver fibrosis^38,39^ and modulates TGFβ1 type 1 receptor (*Alk5*) expression and TGFβ production in renal cell or pancreatic cancer^40,41^. We used siRNA to inhibit *Par2* expression, followed by stimulation with TGFβ1 in LX2 cells, which reduced expression of *Tgfb1* and *Alk5* (Fig. 6g), whereas cells treated with cathepsin G as a Par2 activator showed increased *Alk5* expression (Fig. 6h). As anticipated, *Par2* knockdown reduced phosphorylation of Smad2/3 and Erk1/2 upon TGFβ stimulation compared with that in TGFβ1-treated siControl cells (Fig. 6i), suggesting that Par2 synergistically enhances both canonical and noncanonical TGFβ1 signaling regulated via *Alk5* expression. We next stimulated Par2-knockdown LX2 cells with TGFβ1 and SERPINA3. While treatment with TGFβ1 increased Smad2/3 and Erk1/2 activity, this was partially blocked by SERPINA3 treatment, notably absent in cells pretreated with siPar2 (Fig. 6j). These results demonstrated that SERPINA3 blocks Par2-mediated activation of HSCs by inhibiting the phosphorylation of Smad2/3 and Erk1/2.

#### Pharmacologic Kctd17 inhibition protects from MASH-induced liver fibrosis

**Fig. 7:**
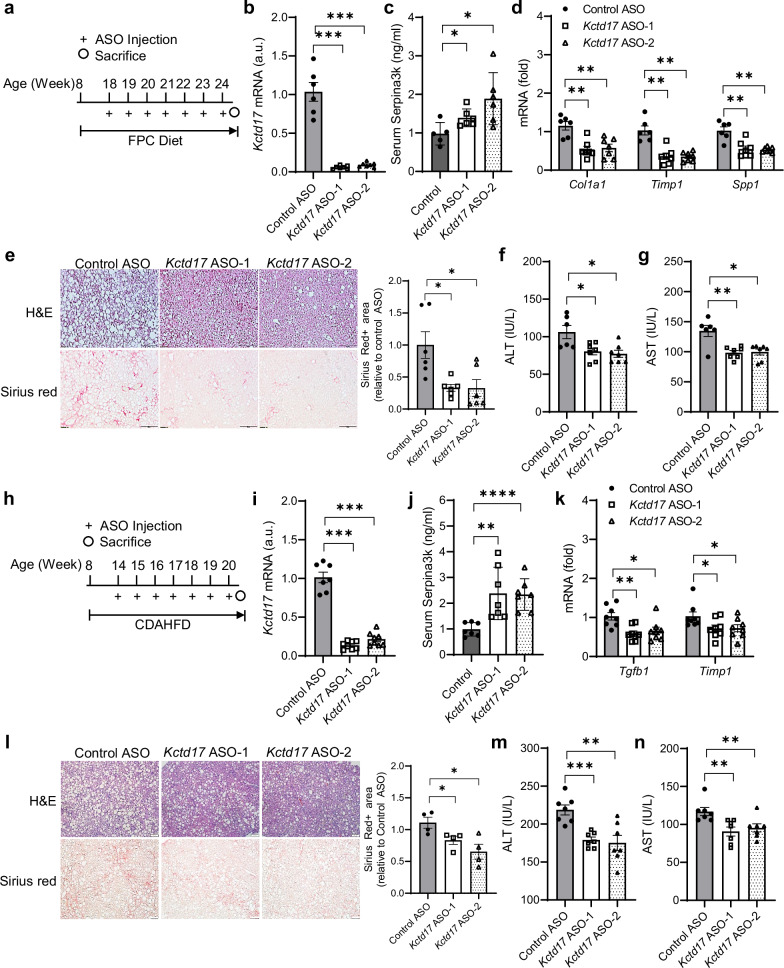
Pharmacologic *Kctd17* inhibition protects from MASH-induced liver fibrosis. **a** C57BL/6 WT mice were fed FPC diet for 10 weeks and then administered control ASO or two different *Kctd17*-targeting ASO (*Kctd17* ASO-1 and ASO-2) weekly by intraperitoneal injection a dose of 25 mg/kg and euthanized 6 weeks after the injections. **b**–**e** Liver *Kctd17* mRNA levels (**b**) Serpina3k protein level in serum (**c**) liver gene expression (*n* = 6–7 per group) (**d**) H&E and Sirius red staining in histological sections of mouse liver, with respective quantification (*n* = 6 per group; scale bar, 100 μm; magnification, 20×) (**e**). **f**, **g** ALT (**f**) and AST (**g**) levels in serum. **h** C57BL/6 WT mice were fed CDAHFD for 6 weeks and then administered control ASO, *Kctd17* ASO-1 or ASO-2 weekly by intraperitoneal injection a dose of 20 mg/kg and euthanized 6 weeks after the injections. **i**–**l** Liver *Kctd17* mRNA levels (**i**), Serpina3k level in serum (**j**) liver gene expression (*n* = 7–8 per group) (**k**) and H&E and Sirius red staining in histological sections of mouse liver, with respective quantification (*n* = 4 per group; scale bar, 100 μm; magnification, 20×) (**l**). **m**, **n** ALT (**m**) and AST (**n**) levels in serum (*n* = 7 per group) in CDAHFD-fed control, *Kctd17* ASO-1-treated or ASO-2-treated mice. *P < 0.05, **P < 0.01, ***P < 0.001 compared with that of the control ASO according to a two-way analysis of variance. All data are shown as the means ± s.e.m.

Finally, we assessed the therapeutic potential of Kctd17 antagonists in treating MASH-induced liver fibrosis. A control ASO and two specific *Kctd17* ASOs (Kctd17 ASO-1, ASO-2)^22^ were administered to FPC diet-fed WT mice (Fig. 7a). Both *Kctd17* ASOs decreased liver *Kctd17* expression and increased serum Serpina3k levels, leading to reduced liver HSC activity (Fig. 7b–d), Sirius red staining and serum ALT and AST levels (Fig. 7e–g), without affecting body weights, liver, eWAT weights or glucose levels compared with the control ASO-treated mice (Supplementary Fig. 7a–d). We replicated these data in CDAHFD-fed mice (Fig. 7h–n and Supplementary Fig. 7e–h). These findings suggest that Kctd17 inhibition can effectively mitigate MASH-induced liver fibrosis, thereby highlighting its potential as a viable therapeutic strategy.

## Discussion

MASH is currently the most prevalent chronic liver disease, significantly contributing to liver-related morbidity and mortality. Recently, the Food and Drug Administration conditionally approved resmetirom, a liver-targeting thyroid hormone receptor agonist, as the first treatment for MASH. It has shown effectiveness in reducing liver fat content and improving hepatic histology without affecting body weight or glucose metabolism^12^. Although this marks a promising new direction in MASH treatment strategies, routine clinical application remains challenging due to the multifactorial nature of liver diseases, which involve complex interaction between metabolic, genetic, and environmental factors.

Previous studies have identified KCTD17 as a significant driver to metabolic dysfunction-associated liver diseases, including MASLD, noting its upregulation in patients with MASH^20,22^. Our study further identified a strong correlation between *KCTD17* expression and disease severity in both human patients and murine models of MASH, indicating that KCTD17 is not only a biomarker of fibrosis but also a potential driver of disease progression. Elevated *KCTD17* levels in the liver of patients with MASH were associated with increased fibrosis, while hepatocyte-specific depletion of *Kctd17* in mice reduced both fibrosis and hepatocellular injury. These results underscore the importance of KCTD17 in MASH pathogenesis, with potential therapeutic implications for targeting this pathway as a means to combat liver fibrosis.

Although increased *KCTD17* levels were consistently observed in both MASH-induced mouse models, a subgroup of patients with MASH or cirrhosis exhibited low *KCTD17* expression. This variability may reflect differences in underlying etiology—such as alcohol-related liver disease versus chronic viral hepatitis—that lead to distinct regulatory mechanisms affecting *KCTD17* expression. It is also possible that the low-*KCTD17* subgroup might represent cases in which compensatory mechanisms are activated in response to liver cell damage and cellular stress during the progression of MASH and cirrhosis, thereby downregulating *KCTD17* in specific hepatic cell populations. Furthermore, individual genetic variations and epigenetic modifications may also influence *KCTD17* levels. This variability raises expectations for uncovering the unknown functions and upstream regulatory mechanisms of *KCTD17* in various liver diseases. Further investigations are warranted to characterize this subgroup in detail and to elucidate the clinical and molecular factors that underlie low *KCTD17* expression in the context of severe fibrosis and cirrhosis.

Serpina3k, as an inhibitor of serine proteases, could have a protective role in multiple diseases. Recent studies have reported that Serpina3k may play a protective role in cardiac ischemia–reperfusion injury and contribute to maintaining energy metabolic balance through the regulation of lactylation^42^. In addition, previous research has shown that tissue-specific Serpina3k exerts anti-apoptotic, antifibrotic, anti-inflammatory and anti-angiogenic effects in renal injury^43^. However, the effects of circulating Serpina3k on extracellular tissue remain poorly understood. In our study, we observed a significant decrease in serum Serpina3k levels, corresponding with reduced hepatic expression in the MASH-induced mouse model. This reduction is expected to influence extrahepatic tissues, where Serpina3k may play a key role in metabolic regulation and cellular homeostasis. Similarly, FGF21, a liver-secreted protein, acts on muscles, adipocytes and the brain to regulate systemic homeostasis and metabolic function^44^. Furthermore, a recent study has demonstrated that liver-specific overexpression of Serpina1 in mice enhances energy expenditure and glucose metabolism by activating mitochondrial UCP1 expression in adipocyte^45^. Therefore, the decrease in serum Serpina3k may not only reflect hepatic dysfunction but also directly impact extrahepatic tissues, potentially affecting inflammation, fibrosis as well as energy metabolism in various tissues.

In humans, SERPINA3 is mainly involved in inflammation and cell death across multiple organs. Reduced SERPINA3 levels in serum are associated with poor survival in patients with liver cancer, and it has been proposed as a biomarker for disease diagnosis and prognosis^46–48^. In line with previous findings, our study observed lower levels of SERPINA3 or Serpina3k in both patients with MASH and mouse models, revealing an inverse relationship between KCTD17 and Serpina3k expression, which contributed to heightened liver fibrosis. Restoring Serpina3k or SERPINA3 levels in mice alleviated fibrosis, reduced HSC activation and lowered cathepsin G activity, suggesting that Serpina3k/SERPNA3 acts as a key effector mediating the antifibrotic effects observed upon Kctd17 inhibition.

*SERPINA3* is transcriptionally activated by Zbtb7b through a putative sequence in its promoter region, significantly impacting both physiological and pathological processes^35^. Notably, Zbtb7b significantly increased *Serpina3k*, while other members of the Serpina3 family remained largely unchanged. However, the direct interaction between Zbtb7b and the promoter elements of Serpina3k requires further investigation. Furthermore, we observed a significant reduction in *Zbtb7b* protein levels in CDAHFD-fed mice (data not shown), reinforcing its potential relevance to MASH progression. To comprehensively understand the pathophysiological functions of Zbtb7b in MASH progression, future in vivo studies should investigate the role of Zbtb7b in hepatocyte-specific contexts by exploring both loss- and gain-of-function scenarios, which may shed light on its contributions to MASH progression.

In conclusion, this study provides crucial insights into the role of KCTD17 in driving MASH-induced liver fibrosis and elucidates the mechanisms behind the reduced expression of Serpina3k/SERPINA3, as well as the functional consequences in MASH-induced mice and patients with MASH (Supplementary Fig. 8). Our findings pave the way for future therapeutic strategies aimed at inhibiting KCTD17 or restoring SERPINA3 expression to combat liver fibrosis in patients with MASH. Future studies should focus on developing specific KCTD17 inhibitors and advancing the clinical translation of SERPINA3-basaed therapies, which could provide a novel and effective treatment approach for liver fibrosis in patients with MASH.

## Supplementary information

Supplementary Information
