# Supplementary material for: Hepatocyte KCTD17-mediated SERPINA3 inhibition determines liver fibrosis in metabolic dysfunction-associated steatohepatitis
Source: Exp Mol Med. 2025 Aug 1;57(8):1673–85. doi: 10.1038/s12276-025-01499-w (PMC12411619; doi:10.1038/s12276-025-01499-w)
Supplement: Supplementary file 1 — Supplementary Information [file 12276_2025_1499_MOESM1_ESM.pdf]

Supplementary Fig. 1

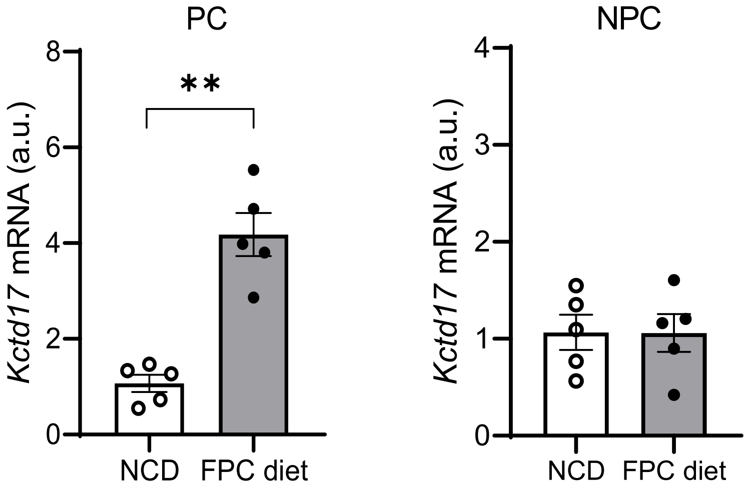

Supplementary Fig. 1. *Kctd17* mRNA expression in various liver cells in mice fed on FPC diet. *Kctd17* mRNA expression in isolated parenchymal cells (PCs) and non-parenchymal cells (NPCs) in C57BL/6 WT mice fed normal chow (NCD) or FPC diet for 4 weeks (n=5 per group). **P* < .05, ***P* < .01 compared with that of the indicated control according to a two-way analysis of variance. All data are shown as the means ± s.e.m.

Supplementary Fig. 2

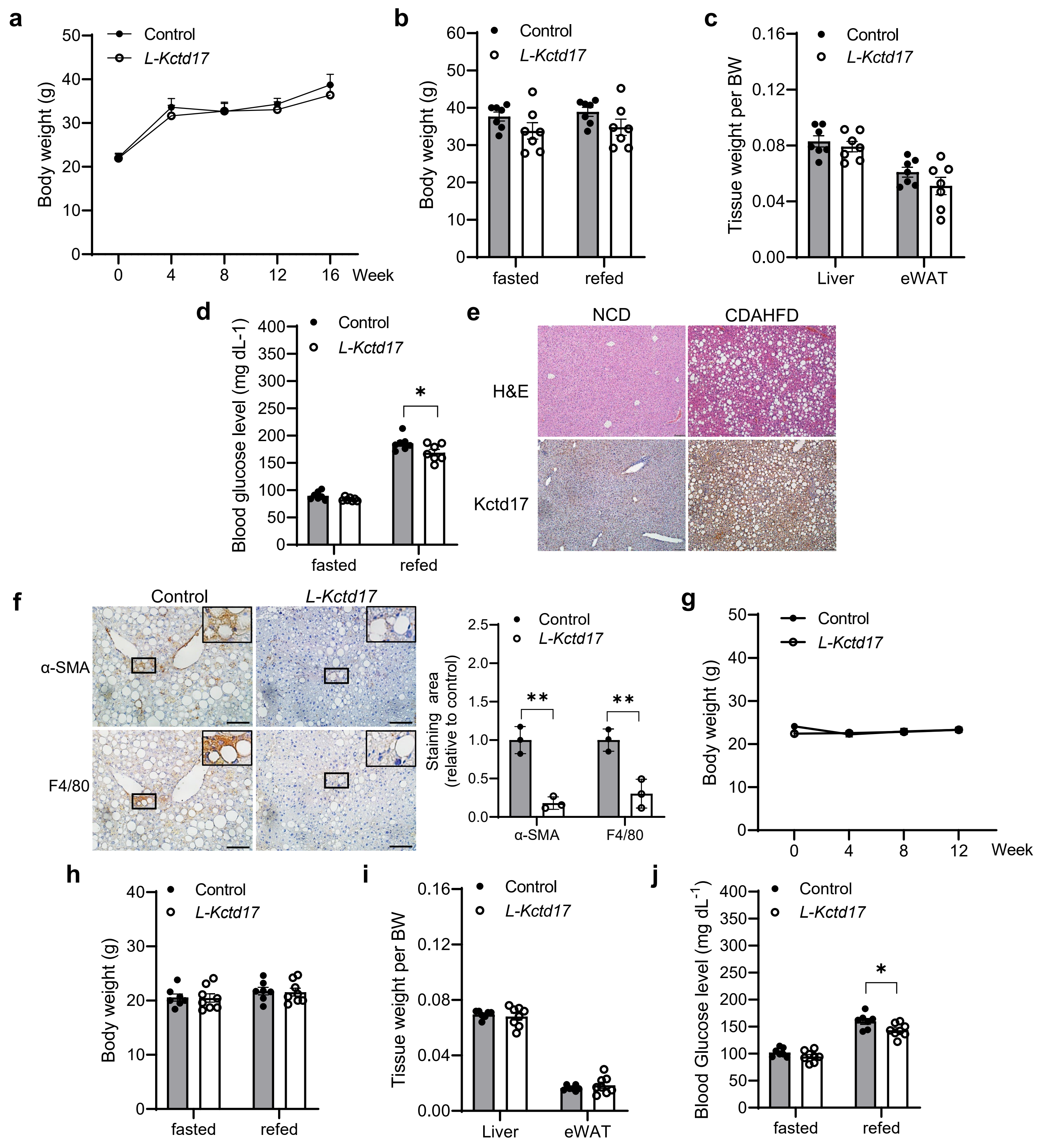

Supplementary Fig. 2. Further characterization of hepatocyte-specific *Kctd17* KO mice fed on MASH-provoking diet. (a and b) Body weight, (c) tissue weight and (d) blood glucose level in hepatocyte-specific *Kctd17* knockout (*L-Kctd17)* mice fed FPC-diet (n=7 per group). (e) H&E staining and immunohistochemistry images of Kctd17 in the liver from CDAHFD-fed mice for 12 weeks (n=4 per group, scale bar: 100 ㎛, magnification: 20x). (f) Immunohistochemistry images of α-Sma and F4/80 of in the liver from CDAHFD-fed control and *L-Kctd17* mice for 12 weeks. (g and h) Body weight, (i) tissue weight and (j) blood glucose level in *L-Kctd17* mice fed CDAHFD. *$P < .05$, ** $P < .01$ compared with that of the indicated control according to a two-way analysis of variance. All data are shown as the means ± s.e.m.

Supplementary Fig. 3

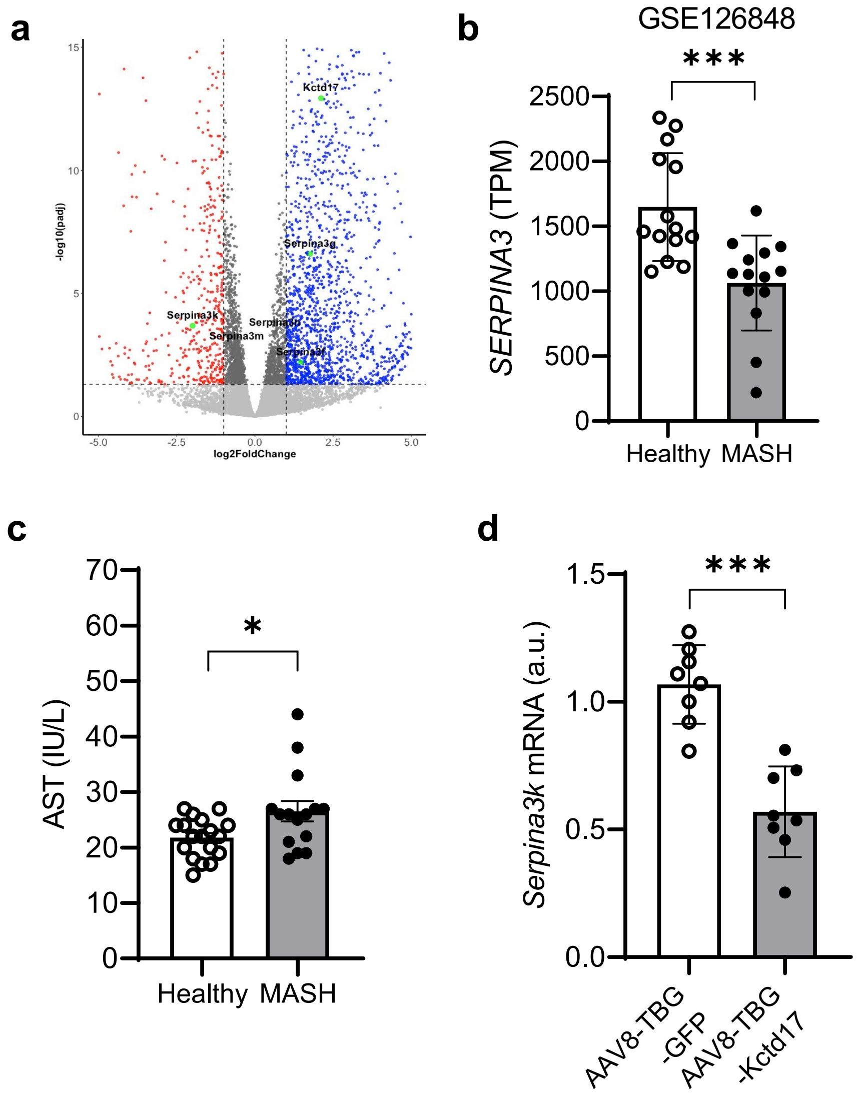

Supplementary Fig. 3. Serpina3k reduced in MASH-induced liver and its secretion is mediated by Kctd17. (a) Volcano plot in RNA-seq database from CDAHFD-fed C57BL/6 WT mice (GSE138449, n=4 per group). (b) SERPINA3 expression levels in RNA-Seq database in livers from Healthy and patients with MASH (GSE126848, Healthy=14, MASH=14). (c) AST level in healthy subjects and patients with MASH. (d) *Serpina3k* mRNA level in AAV8-TBG-Kctd17 mice (n=8). **P* < .05, ***P* < .01, ****P* < .001 compared to that of the indicated control according to a two-way analysis of variance. All data are shown as the means ± s.e.m.

Supplementary Fig. 4

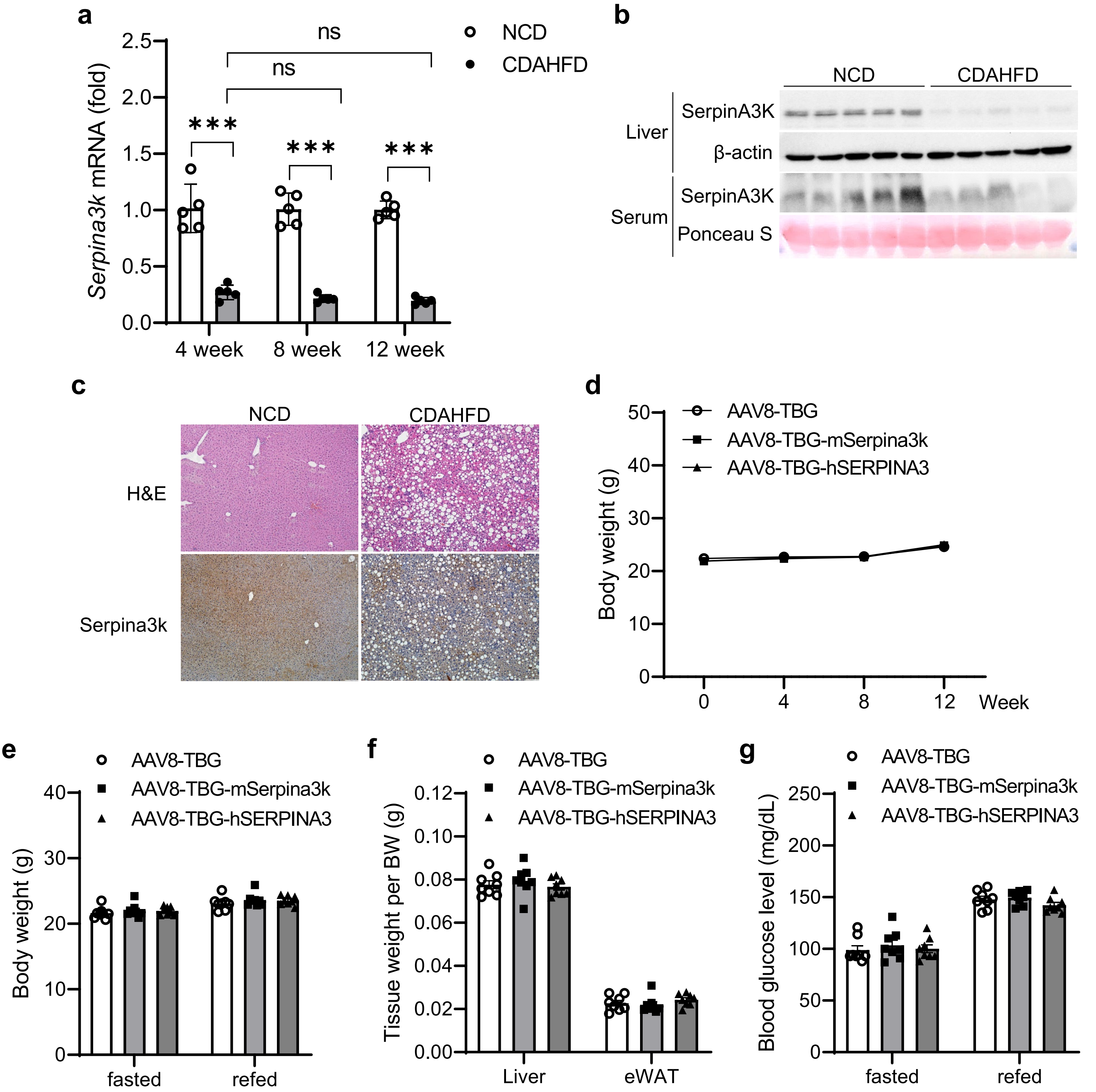

Supplementary Fig. 4. Further characterization of hepatocyte-specific Serpina3k/SERPINA3 mice fed on CDAHFD. (a) *Serpina3k* mRNA expression in the liver, (b) protein level in the liver and serum (n=5 per group) and (c) H&E and immunohistochemical staining imaging of Serpina3k in liver tissue from CDAHFD-fed WT mice for 4 weeks (n=4 per group, scale bar: 100 ㎛, magnification: 20x). (d and e) Body weight, (f) tissue weight and (g) blood glucose level in of hepatocyte-specific Serpina3k or SERPINA3 mice (AAV8-TBG-mSerpina3k or AAV8-TBG-hSERPINA3) fed CDAHFD (n=7-8 per group). ***P < .001 compared with that of the indicated control according to a two-way analysis of variance. All data are shown as the means ± s.e.m.

Supplementary Fig. 5

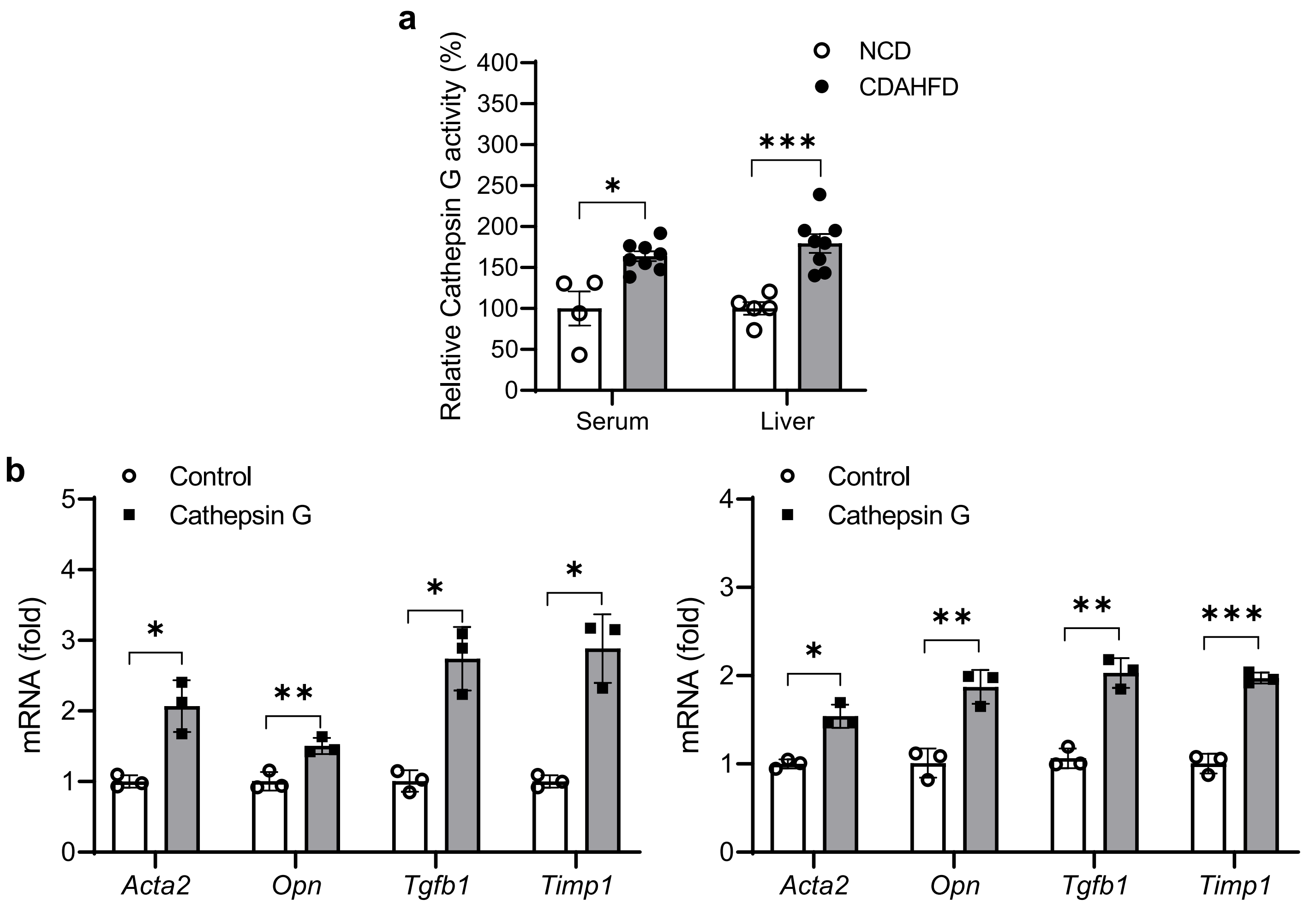

Supplementary Fig. 5. Cathepsin G activity in CDAHFD-fed mice and fibrotic effects in HSCs. (a) Relative cathepsin G activity in serum and liver from CDAHFD-fed mice (n=4-8 per group). (b) Fibrogenic gene expression by treatment of cathepsin G (100 ng/ml) for 24 hours in HSC-T6 (left) and LX2 (right) cells. $^*P < .05$, $^{**}P < .01$, $^{***}P < .001$ compared to that of the indicated control according to a two-way analysis of variance. All data are shown as the means ± s.e.m.

Supplementary Fig. 6

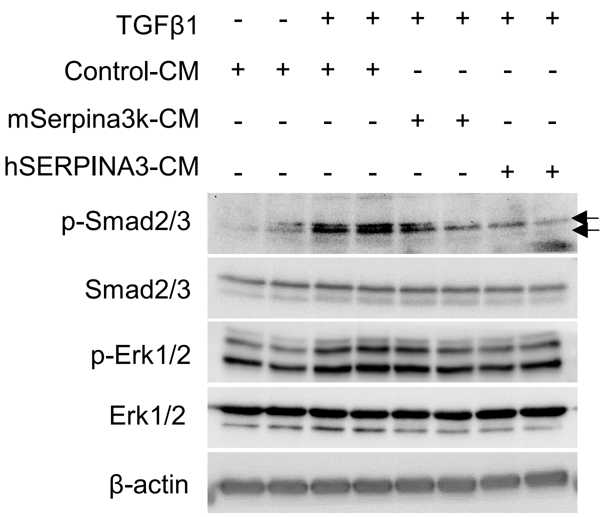

Supplementary Fig. 6. Serpina3k/SERPINA3 blocks TGFβ1-mediated p-Smad2/3 and p-Erk1/2. Western blots of pSmad2/3, pErk1/2 from HSC-T6 cells with co-treatment of Control, mSerpina3k- or hSERPINA3-CM with TGFβ1 (10 ng/ml) for 1 hour.

Supplementary Fig. 7

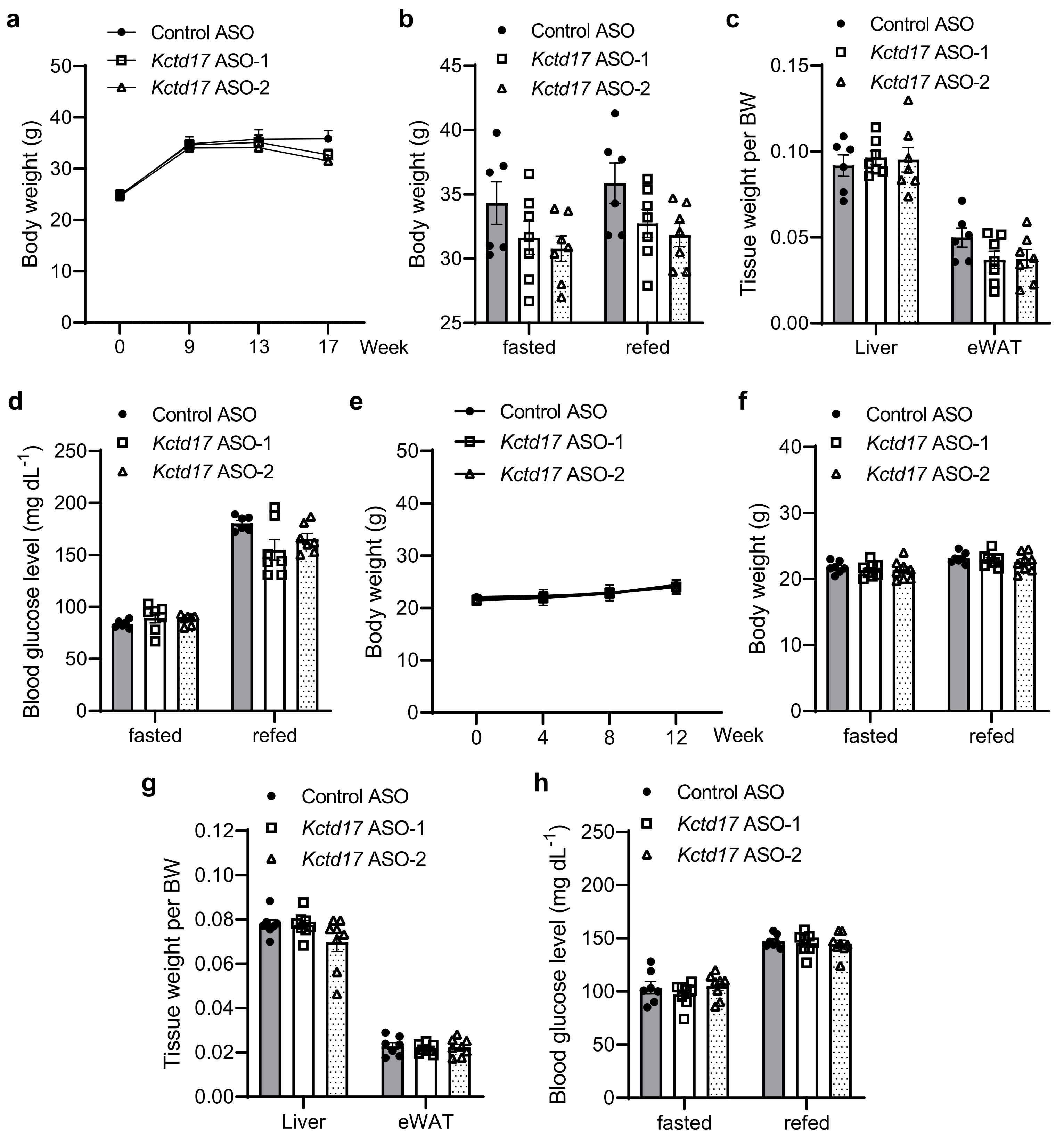

Supplementary Fig. 7. Further characterization of liver-directed *Kctd17* ASO-treated mice fed on MASH-provoking diet. (a and b) Body weight, (c) tissue weight and (d) blood glucose level in FPC diet-fed Control, *Kctd17* ASO-1 or -2 mice (n=6-7 per group) mice. (e and f) Body weight, (g) tissue weight and (h) blood glucose levels in CDAHFD-fed Control, *Kctd17* ASO-1 or -2 mice (n=7-8 per group) mice. Statistical significance according to a two-way ANOVA. All data are shown as the means ±s.e.m

Supplementary Fig. 8

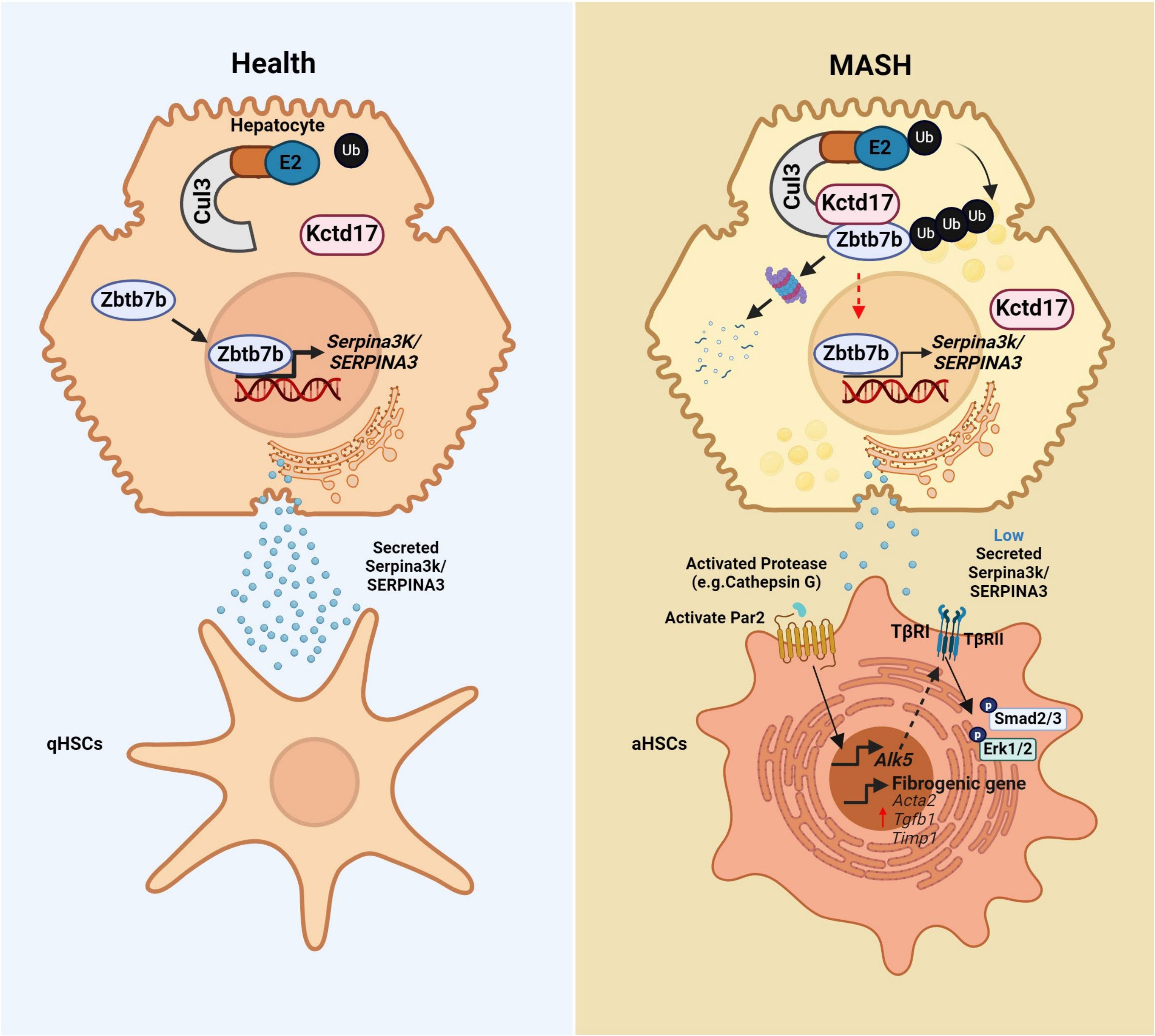

Supplementary Fig. 8. Model representing the effect of KCTD17-SERPINA3 axis in MASH-induced liver fibrosis

Supplementary Table 1. Quantitative PCR Primer Sequences

Primer	Forward	Reverse
m36b4	AGATGCAGCAGATCCGCAT	GTTCTTGCCCATCAGCACC
h36b4	CGACCTGGAAGTCCAACTAC	ATCTGCTGCATCTGCTTG
hKctd17	AAACCAAGAGCACGGAGG	GAGGGAGAAAAGGTTAGCGG
sgKctd17	GGCACGGTGTTCCTGAC C	CTCATCCCTGTCCGATTGAAGC
mKctd17	GAGCTCACACAGATGGTATCC	TGGTCCTCACTCCCATAGTT
mActa2	ATGCTCCCAGGGCTGTTTTCCCAT	GTGGTGCCAGATCTTTTCCATGTCG
mCtgf	ACTGCCCCTTCCCGAGAA	ACTGCCCCTTCCCGAGAA
mCol1a1	GCTCCTCTTAGGGGCCACT	CCACGTCTCACCATTGGGG
mOpn	CTGACCCATCTCAGAAGCAGAATCT	TCCATGTGGTCATGGCTTTCATTGG
mTimp1	CTCAAAGACCTATAGTGCTGGC	CAAAGTGACGGCTCTGGTAG
mTgb1	GACCGCAACAACGCCATCTA	GGCGTATCAGTGGGGGTCAG
mSpp1	CTGACCCATCTCAGAAGCAGAATCT	TCCATGTGGTCATGGCTTTCATTGG
hCol1a1	GATTCCCTGGACCTAAAGGTGC	AGCCTCTCCATCTTTGCCAGCA
hTgfb1	TACCTGAACCCGTGTTGCTCTC	GTTGCTGAGGTATCGCCAGGAA
hTimp1	GGAGAGTGTCTGCGGATACTTC	GCAGGTAGTGATGTGCAAGAGTC
mSerpina3k	CCAGCCAAATAGAGGAGCTAAA	TAGGTCAGCTTGTTCTGTGAAG
mSerpina3g	GCATCAGGGAAGTCTTCTCCAC	CACAACATCCGACACCTGCCAT
mSerpina3m	AGCAGGTGGAAGCCAGCTTACA	CAGCTCTGGAAGGATGTCCTTC
mSerpina3n	CAACCAGAGACCCTGAGGAAGT	AGGACATCCTCCAGGCTGTAGT
mSerpina3f	ACTACAGCCTGGAGCACATCCT	ACATCCAGCACAGCCTTGTGGA
hSerpina3	TGCCAGCGCACTCTTCATC	TGTCGTTCAGGTTATAGTCCCTC
mSerpina3k (exo)	TTGCCTTTCTCTCCACAGGT	CAGGACAGATTCCAGCCATT
mZbtb7a	TGCGAGAAGGTGATTCAGGGTG	TTCCGCATGTGCACCTTCAGCT
mZbtb7b	CACACTGGTGAGAAGCCCTTTG	GTTCTCCTGTGTGCTTCCGCAT
hPar1	GTGATTGGCAGTTTGGGTCT	GCCAGACAAGTGAAGGAAGC
hPar2	CCTGGCCATGTACCTGATCT	GACACTTCGGCAAAGGAGAG
rPar1	GCCAGAAGCACCTTTACAGC	TTCAGGTGGCTAGAGCAGGT
rPar2	GAACATCACCACCTGTCACG	TCATGAGCACGTAGGCAGAC
